# Direct Homo- and Hetero-Interactions of MeCP2 and MBD2

**DOI:** 10.1371/journal.pone.0053730

**Published:** 2013-01-15

**Authors:** Annette Becker, Lena Allmann, Maria Hofstätter, Valentina Casà, Patrick Weber, Anne Lehmkuhl, Henry D. Herce, M. Cristina Cardoso

**Affiliations:** 1 Department of Biology, Technische Universität Darmstadt, Darmstadt, Germany; 2 Max Delbrück Center for Molecular Medicine, Berlin, Germany; Université Paris-Diderot, France

## Abstract

Epigenetic marks like methylation of cytosines at CpG dinucleotides are essential for mammalian development and play a major role in the regulation of gene expression and chromatin architecture. The methyl-cytosine binding domain (MBD) protein family recognizes and translates this methylation mark. We have recently shown that the level of MeCP2 and MBD2, two members of the MBD family, increased during differentiation and their ectopic expression induced heterochromatin clustering *in vivo*. As oligomerization of these MBD proteins could constitute a factor contributing to the chromatin clustering effect, we addressed potential associations among the MBD family performing a series of different interaction assays *in vitro* as well as *in vivo*. Using recombinant purified MBDs we found that MeCP2 and MBD2 showed the stronger self and cross association as compared to the other family members. Besides demonstrating that these homo- and hetero-interactions occur in the absence of DNA, we could confirm them in mammalian cells using co-immunoprecipitation analysis. Employing a modified form of the fluorescent two-hybrid protein-protein interaction assay, we could clearly visualize these associations in single cells *in vivo*. Deletion analysis indicated that the region of MeCP2 comprising amino acids 163–309 as well the first 152 amino acids of MBD2 are the domains responsible for MeCP2 and MBD2 associations. Our results strengthen the possibility that MeCP2 and MBD2 direct interactions could crosslink chromatin fibers and therefore give novel insight into the molecular mechanism of MBD mediated global heterochromatin architecture.

## Introduction

The epigenetic information in mammals is encoded in the form of methylated cytosines at the C5 position within CpG dinucleotides and modifications of histone tails among many others. Methylated CpGs (mCpGs) are recognized by methyl-cytosine binding proteins like Kaiso proteins, SRA domain containing proteins and methyl-cytosine binding domain (MBD) proteins [1]. They read this epigenetic DNA mark and translate it into higher-order chromatin structures and therefore form an important link between DNA methylation and chromatin organization.

MBD1, MBD2, MBD3, MBD4 and MeCP2 represent the most studied members of the MBD protein family [2]. They all bear a conserved MBD domain that enables them, except for MBD3, to bind to mCpGs [3]–[6] leading to their predominant accumulation at constitutive heterochromatin *in vivo* [4], [7].

MeCP2 was originally described as a transcriptional regulator imposing local repressive chromatin structures through recruitment of histone-modifying enzymatic activities [8]–[15]. Recent reports further implicate the intrinsic capability of MeCP2 to organize global heterochromatin architecture [7], [16], [17]. We have shown that MeCP2 induces large-scale chromatin reorganization *in vivo* - in particular clustering of pericentric heterochromatin - in a dose-dependent manner [7]. The MBD was shown to be necessary and sufficient for MeCP2 chromatin aggregation ability, and a MeCP2 deletion construct lacking the NH_2_-terminal region and the MBD is insufficient to induce clustering of chromatin in vivo [7]. Mutations within the *MECP2* gene have been linked to the neurological disease Rett Syndrome [18], [19]. We recently tested several missense mutations within MeCP2 MBD and found that they affect MeCP2 ability to accumulate at pericentric heterochromatin and/or cluster heterochromatin *in vivo* [20], [21].

In line with the role of MeCP2 in pericentric chromatin clustering *in vivo*, *in vitro* assays demonstrated that MeCP2 can compact polynucleosomes into highly condensed suprastructures [16], [22]. Nucleosome interaction studies indicated that mostly residues in the COOH-terminal regions of MeCP2 are involved in chromatin binding [22], [23]. Importantly, maximal compaction of nucleosomal arrays involving secondary and tertiary chromatin structures does not take place in the absence of the region COOH-terminal from the MBD [16].

As a potential mechanism underlying MeCP2 coordination of global chromatin architecture, a sandwich-like formation of MeCP2 with nucleosomes and/or DNA has been proposed, most probably requiring at least two chromatin or DNA binding sites within MeCP2 [16], [22]. Oligomerization of MeCP2, resulting in nucleosome-MeCP2-MeCP2-nucleosome or DNA-MeCP2-MeCP2-DNA complexes, has also been proposed [7], [16]. The fact that MeCP2 induces different levels of chromatin structure *in vitro* depending on the ratio of MeCP2 to nucleosomes, together with the *in vivo* findings that increasing level of MeCP2 results in increased heterochromatin aggregation strengthen this hypothesis [7], [16]. Recent studies illustrated inter-domain associations of certain domains of MeCP2 *in trans* using fluorescence anisotropy and circular dichroism [24]. Furthermore, using atomic force microscopy, MeCP2 has been shown to exist as a monomer and dimer on DNA [25]. However, hydrodynamic studies describing recombinant MeCP2 as a monomer have challenged oligomerization of MeCP2 [26], [27]. We therefore assayed potential interactions of MeCP2 with itself as well as other members of the MBD protein family that could be involved in crosslinking heterochromatin fibers. Using *in vitro* pull-down experiments, we show that MeCP2 indeed forms direct homo-interactions with itself and hetero-associations to MBD2. We further mapped the interacting domains and found one defined region of MeCP2 and of MBD2 mediating both, binding to MeCP2 and MBD2. Finally, we could confirm these associations *in vivo* using different interaction assays.

## Materials and Methods

### Expression Plasmids

Mammalian expression vectors for rat MeCP2G, MeCP2R and GBP-laminB were described before [7], [28].

MeCP2R was used to create the vector pmRFP-N2 by replacing EGFP from pEGFP-N2 (Clontech; Mountain View, CA, USA) with mRFP using BamHI and BsrGI.

Mouse MBD2 tagged with GFP (MBD2G) was created by replacing EGFP from pEGFP-N1 (Clontech; Mountain View, CA, USA) with the MBD2-GFP fragment from the pFastBac vector MBD2aG (described below) using EcoRI and BsrGI.

MBD2 tagged with RFP (MBD2R) was created by subcloning MBD2 from the pFastBac vector MBD2aG with EcoRI and NotI into pBluescript KS+ (Stratagene; Agilent Technologies Genomics, Santa Clara, CA, USA) cut with EcoRI and PspOMI. MBD2 was then transferred into pmRFP-N2 using EcoRI and KpnI digestion.

To produce pMeCP2R.9, pMeCP2G.9 [29] was digested with BamHI and NheI releasing the MeCP2.9 fragment. The insert was then ligated into pmRFP-N2 vector, cut before with the same restriction sites.

pMBD2R.1 for expression in mammalian cells was created by cloning the EcoRI/NotI fragment of pFB-MBD2R.1 into pEGFP-N1, thereby replacing EGFP by cutting with the same enzymes.

For expression in Sf9 cells (Invitrogen; Paisley PA4 9RF, UK) the Bac-To-Bac baculovirus expression system (Invitrogen; Paisley PA4 9RF, UK) was used. Full length MeCP2 constructs tagged with GFP (MeCP2G) and strep (stMeCP2) were described before [29].

For construction of MeCP2 tagged with mRFP (MeCP2R), GFP was replaced from the pFastBac vector MeCP2G by mRFP using PspOMI and XhoI sites. Vectors for GFP expression, pFB-C-GFP and pFB-C-GFP octa, were created by amplification of GFP from pEGFP-C1 (Clontech; Mountain View, CA, USA) with NotI and XbaI sites and cloned into pFastBac-1 (Invitrogen; Paisley PA4 9RF, UK). AsiSI and NotI sites were introduced by oligo cloning resulting in pFB-C-GFP-octa.

For mRFP expression, pFB-mRFP was created by cloning the EcoRI-NotI fragment from pmRFP-N2 into pFastBac-1.

Mouse MBD1a, MBD2a, MBD3 and MBD4 tagged with GFP were created by amplification from already described mammalian expression constructs [4] with primers including SalI and NotI sites and cloned into pFB-C-GFP. Strep-tagged MBD2 (MBD2st) was created by replacing GFP from MBD2G with a strep-tag [29], amplified using primers flanked by NotI and XhoI sites.

MBD2 tagged with mRFP (MBD2R) was taken from the mammalian expression vector using EcoRI and NotI and cloned into pFastBac-1.

The MeCP2 deletion constructs MeCP2Y.3 (aa 1–162) and MeCP2Y.5 (aa 77–162) were created from mammalian expression vectors described before [7] by cloning the NotI or BamHI-NotI fragment into pFastBac-1, respectively.

MeCP2G.8 (aa 310–492) and MeCP2G.9 were amplified from mammalian expression vector pMeCP2G.6 [30] with primers including BglII and NotI or BamHI and NotI sites, respectively. MeCP2G.8 BglII-NotI fragment was ligated into BamHI and NotI sites in pFastBac-1. MeCP2.9 BamHI-NotI fragment was ligated into pFB-C-GFP cut with same enzymes. For mCherry-tagged MeCP2.9, GFP was replaced with mCherry by ligating PspOMI-mCherry-XbaI fragment with pFB-MeCP2G.9 cut with NotI and XbaI resulting in MeCP2Ch.9.

MeCP2G.10 was custom synthesized into pCR4-TOPO (Invitrogen; Paisley PA4 9RF, UK) flanked by SalI and NotI sites (Entelechon; Bad Abbach, Germany). The fragment was ligated into pFB-C-GFP, cut with the same sites.

MBD2 deletion constructs MBD2.1 (aa 1–152) and MBD2.2 (aa 153–414) were amplified from pFB-MBD2a-C-GFP (MBD2G) with forward primers carrying AsiSI sites and reverse primers with NotI sites. Digested fragments were ligated into pFB-C-GFP octa cut with the same enzymes. For generation of RFP-tagged MBD2.1, pFB-MBD2G.1 was digested with EcoRI and NotI and the fragment was ligated into pFB-mRFP cut with EcoRI and PspOMI. MBD2.3 (aa 222–414) and MBD2.4 (aa 236–414) were amplified from pFB-MBD2G.2 with forward primers coding for SalI and reverse primers keeping the existing NotI restrictions sites and then cloned into pFB-C-GFP octa. GFP was then replaced by mcherry.MBD2.5 (aa 153–221) was synthesized (in pMK-RQ; GeneArt; Invitrogen; Paisley PA4 9RF, UK) flanked by EcoRI and PspOMI sites and cloned into pFB-mRFP resulting in MBD2R.5. MBD2.6 (aa 153–235) was synthesized (in pMK-RQ; GeneArt; Invitrogen; Paisley PA4 9RF, UK) flanked by SalI and NcoI and cloned into pFB-C-GFP octa. GFP was then replaced by mRFP from pFB-mRFP using AgeI and XhoI to create MBD2R.6.

### Cell Culture, Transfection and Staining

HEK 293-EBNA cells (Invitrogen; Paisley PA4 9RF, UK) were cultured and transfected as described [30].

C2C12 mouse myoblasts [31] were grown at 37°C and 5% CO_2_ in Dulbecco’s Modified Eagle Medium (DMEM) supplemented with 20% fetal calf serum. Cells were grown to 70% confluency on 16 mm glass cover slips in 6 well plates and transfected using poly-ethylenimine (PEI, 1 mg/ml in ddH_2_O, pH 10; Sigma-Aldrich, St. Louis, MO, USA) [21]. One day after transfection, the cells were fixed using a formaldehyde (FA) gradient as described before [21]. After fixation, cells were mounted in Vectashield antifade medium (Vector Laboratories, Burlingame, CA, USA).

Sf9 insect cells (Invitrogen; Paisley PA4 9RF, UK) were cultivated in EX-CELL 420 Insect Serum Free (SAFC) medium supplemented with 10% fetal calf serum. The culture was kept shaking at 100 rpm and 28°C. Transfection of Sf9 cells to produce recombinant baculovirus was performed using Cellfectin (Invitrogen; Paisley PA4 9RF, UK) according to the manufacturer’s instructions. For protein production, Sf9 insect cells were infected with the recombinant baculovirus (P3 stock) and incubated for 5 days shaking at 28°C. The cells were pelleted by centrifugation (200×g, 5 min, 4°C).

### Fluorescent 3-hybrid Assay

Fixed C2C12 cells were examined with a Zeiss Axioplan2 wide-field epifluorescence microscope. To quantify the % of cells displaying co-localization, 100 cells were analyzed per transfection. Image stacks (0.6 µm Z interval) were acquired using a 63×Plan-Apochromatic NA 1.4 oil immersion phase contrast objective lens and a PCO Sensicam QE cooled CCD camera.

### *In vivo* Co-immunoprecipitation Assays

For co-immunoprecipitation analysis of full-length MBD proteins, HEK 293-EBNA cells (p100 dish) co-transfected with GFP- and RFP- fused MBD proteins or GFP control were pelleted after washing with 1×PBS and resuspended in 200 µl 4×PBS and incubated on ice for 5 min. For efficient lysis, incubation was followed by a short syringe treatment (2–3 times) and subsequent dilution of the lysate with 600 µl buffer A (20 mM Tris-HCl pH 8, 0.5% NP-40, 0.5 mM EDTA) to obtain a NaCl concentration of 137 mM or 200 µl buffer A for a final concentration of 274 mM NaCl. After incubation for 15 min on ice and centrifugation at 20.000×g for 12 min at 4°C, the lysate was incubated with GFP binding protein (GBP) coupled to sepharose beads (GFP-Trap, ChromoTek, Planegg-Martinsried, Germany; [28]) for one hour at 4°C on a rotary shaker. Afterwards, the beads were washed three times with 300 µl buffer B1 (20 mM Tris-HCl pH 7.5, 200 mM NaCl, 0.5% NP-40, 0.5 mM EDTA) or buffer B2 (20 mM Tris-HCl pH 8, 275 mM NaCl, 0.5 mM EDTA, 0.1% NP-40) and then resuspended in 30 µl 1×SDS-containing sample buffer and boiled for 6 min at 99°C to be analyzed by SDS-PAGE followed by Western Blot. For co-immunoprecipitation analysis of domains of the MBD proteins, HEK 293-EBNA cells were co-transfected with plasmids coding for RFP- and GFP-labeled domains of the MBD protein or GFP control. The co-transfected cells were lysed using buffer A (20 mM Tris-HCl pH 8, 0.5% NP-40, 0.5 mM EDTA) supplemented with 200 mM NaCl. After syringe treatment (2–3 times), the lysate was incubated on ice for 15 min followed by centrifugation at 20.000×g for 12 min at 4°C. Afterwards, the lysate was incubated for one hour at 4°C together with GBP-coupled sepharose beads. The immobilized protein complexes were washed with buffer A supplemented either with 200 mM NaCl or 300 mM NaCl and resuspended in 40 µl 1×SDS-containing sample buffer and boiled for 6 min at 99°C to be analyzed by SDS-PAGE followed by Western Blot. All buffers were supplied with protease inhibitors in following concentrations: AEBSF 1 mM (AppliChem; Darmstadt, Germany), Leupeptin 1 mM (Sigma-Aldrich, St. Louis, MO, USA), Pepstatin A 1 nM (Sigma-Aldrich, St. Louis, MO, USA) and Aprotinin 2 ng/ml (Sigma-Aldrich, St. Louis, MO, USA).

### *In vitro* Binding Assays

Sf9 cells overexpressing the protein of interest were resuspended in buffer C (25 mM Tris-HCl, pH 8.0, 1 M NaCl, 50 mM glucose, 10 mM EDTA, 0.2% Tween-20, 0.2% NP-40) or D (25 mM Tris-HCl, pH 7.5, 500 mM NaCl, 5% Glycerol, 0.2% Triton X-100). All buffers used for the in vitro assays were supplemented with the protease inhibitors AEBSF, Leupeptin, Pepstatin and Aprotinin in the concentrations as described above. After incubation on ice for 10 min, cells were disrupted by sonification. After centrifugation (20.000×g, 4°C) for 30 min, the supernatant was removed from the cell debris and used for the next steps. The GFP or RFP- fused proteins were immobilized to GBP or RFP binding protein (RBP) coupled to sepharose beads (GFP-Trap, RFP-Trap, ChromoTek, Planegg-Martinsried, Germany) [28], [32] by incubation at 4°C for 30 min. Afterwards, the immobilized proteins were washed three times with buffer C or D followed by two washes using the interaction buffer E (PBS, 0.05% NP-40) with varying amount of NaCl as indicated in the legends to each figure.

Purification of strep-tagged recombinant proteins was achieved by incubating the protein extract with 500 µl of Strep-Tactin Sepharose (IBA GmbH; Goettingen, Germany) beads for three hours at 4°C on a rotary shaker. For elution of the strep-tagged proteins, the beads were incubated with D-Desthiobiotin (0.5 mg/ml; IBA), dissolved in 1x PBS, for 30 min at 4°C. After centrifugation (200×g, 2 min), beads were separated from the eluate containing the purified proteins. The first two eluates were pooled and used for the assay.

For *in vitro* binding assays, immobilized recombinant GFP- or RFP- tagged proteins as indicated were incubated with nearly equal amounts of purified proteins or protein extracts in 500 µl buffer E for one hour at 4°C on a rotary shaker. After a short spin, the beads were washed three times with buffer E, dissolved in 40 µl 1×SDS-containing sample buffer and boiled for 10 min at 99°C.

### Western Blot Analysis

Western blotting was performed as described before [33] transferring the proteins on a nitrocellulose membrane (GE Healthcare, München, Germany). Visualization of the immunoreactive bands was achieved by ECL plus Western Blot Detection reagent (GE Healthcare; München, Germany). The following antibodies were used: rat monoclonal anti GFP 3H9 (ChromoTek, Planegg-Martinsried, Germany), rabbit polyclonal anti RFP, mouse monoclonal anti GFP (Roche; Mannheim, Germany) and rat monoclonal anti RFP 5F8 [34] (ChromoTek, Planegg-Martinsried, Germany). Horseradish peroxidase conjugated (HRP) anti mouse IgG (GE Healthcare; München, Germany), HRP conjugated goat anti rat IgG (Jackson; West Grove, PA, USA) and HRP conjugated anti rabbit IgG (Sigma-Aldrich, St. Louis, MO, USA) were used as secondary antibodies. To detect strep-fused proteins, the membrane was incubated with horseradish peroxidase conjugated StrepTactin (Bio-Rad Laboratories, Hercules, CA, USA) for 1.5 hours at room temperature.

## Results and Discussion

### MBD2 and MeCP2 Exhibit the Strongest Interactions among all MBD Proteins

Based on our recent observations that ectopic expression of MeCP2 induces clustering of pericentric heterochromatin *in vivo* [7], we hypothesized that MeCP2 could potentially interact with itself and accomplish the chromatin aggregation process not only as a monomer, but also as di- or oligomer. Besides MeCP2-MeCP2 homo-interactions, also MeCP2 hetero-interactions to other MBD family members could be an additional factor contributing to MeCP2 mediated large-scale heterochromatin organization.

**Figure 1:**
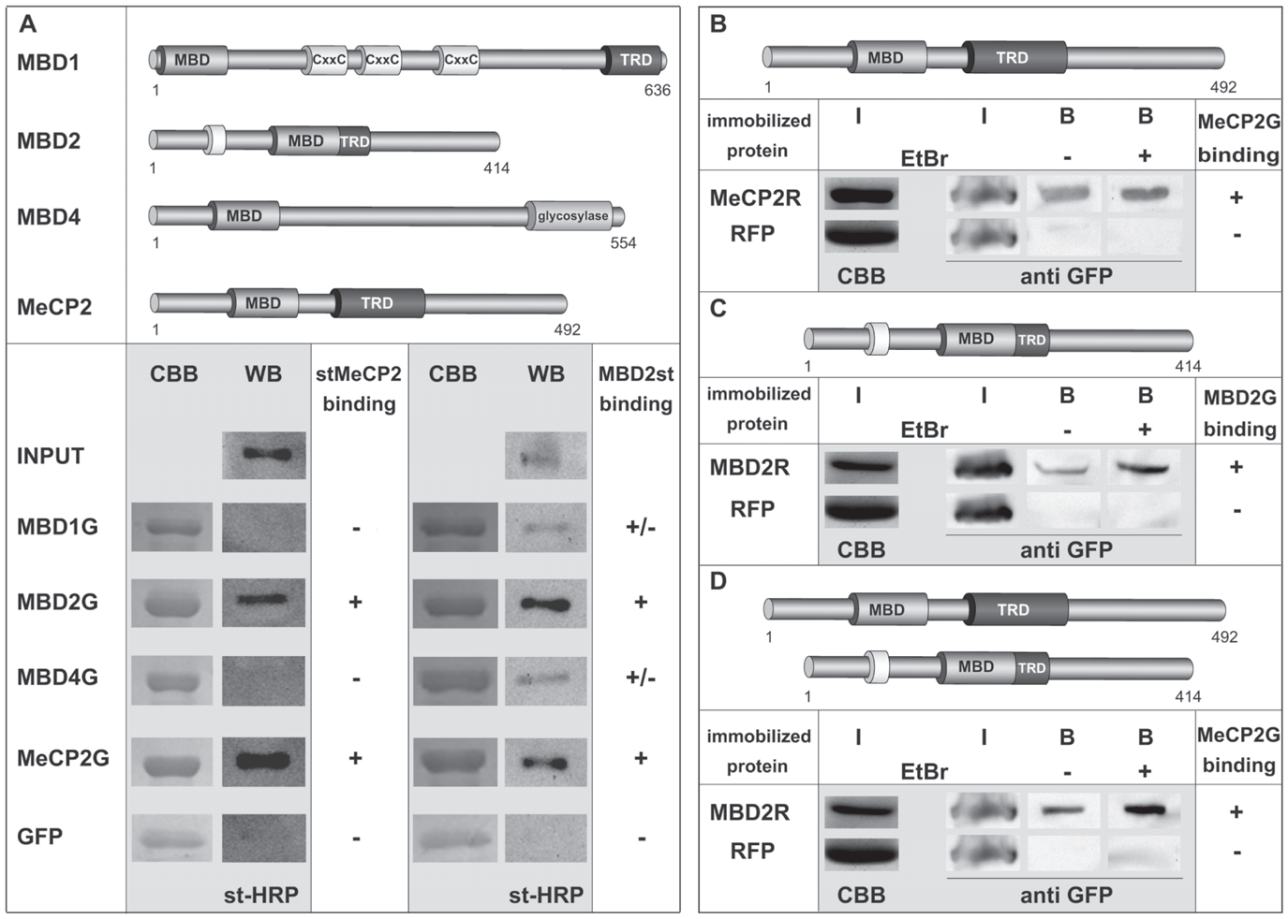
Interactions among MBD proteins. (**A**) Upper panel: schematic representation of methyl-cytosine binding domain (MBD) proteins. The numbers stand for amino acid coordinates. (MBD) methyl-cytosine binding domain, (TRD) transcriptional repression domain, (CxxC) cysteine rich domain. Lower panel: In vitro pull down experiments using purified strep (st) fused MeCP2 or MBD2 and GFP-labeled full-length (fl) MBD proteins immobilized to GFP-binding protein (GBP) bound sepharose beads. The proteins were extracted using 0.5 M NaCl containing lysis buffer. The interaction assays were performed either in PBS supplemented with 0.05% NP-40 (stMeCP2) or in PBS plus 150 mM NaCl and 0.05% NP-40 (MBD2st). Interacting st-taged fl MeCP2 and MBD2 were assessed by Western blot using st-HRP conjugate. Coomassie Brilliant Blue (CBB) staining of the SDS-gel after protein transfer shows GFP-labeled immobilized MBD proteins used for the pull-down assay. (**B, C, D**) Interactions between MeCP2 and MBD2 are not bridged by DNA. *In vitro* pull-down experiments were performed using immobilized RFP-fused MeCP2 (MeCP2R) or MBD2 (MBD2R) and GFP-labeled MeCP2 (MeCP2G) or MBD2 (MBD2G) either with or without addition of ethidium bromide (EtBr; 10 µg/ml). All proteins were extracted in 1 M NaCl containing lysis buffer. In the case of MeCP2 homo-interactions (**B**), the interaction was performed in PBS plus 0.05% NP-40 buffer. For the homo-interactions of MBD2 (**C**), PBS was additionally supplemented with 110 mM NaCl and 0.05% NP-40, and for the hetero-binding of MBD2 and MeCP2 (**D**), PBS plus 125 mM NaCl and 0.05% NP-40 was used. (**B, C, D**) For input control (I), ¼ of the protein amount used for the interaction assay of the immobilized RFP-tagged proteins was loaded on SDS-PAGE and stained with CBB. Also ¼ of the GFP-tagged proteins used for the pull-down were visualized by western blot using anti GFP (I). Interacting GFP-fused MeCP2 or MBD2 (B) were assessed by western blot using anti GFP antibody.

For this reason, we set out to analyze interactions of MeCP2 with itself and other members of the MBD protein family and performed *in vitro* pull down experiments using recombinant proteins produced in Sf9 insect cells. Immobilized GFP-tagged MeCP2 (MeCP2G), MBD1 (MBD1G), MBD2 (MBD2G), MBD4 (MBD4G) and GFP alone were incubated with full-length strep-fused MeCP2 (stMeCP2). SDS-PAGE followed by western blot analysis using strep-HRP (st-HRP) conjugate revealed that stMeCP2 exhibited binding to itself as well as MBD2G, but not to MBD1G, MBD4G and GFP (Figure 1A and Figure S1A). Prompted by the result, that stMeCP2 strongly interacted with MBD2G, we went on analyzing the binding ability of strep-fused MBD2 (MBD2st) to itself as well as to MeCP2G, MBD1G, MBD4G and GFP. Whereas GFP alone, MBD1G and MBD4G showed very weak to no binding to MBD2st, MBD2G as well as MeCP2G again exhibited the strongest association to MBD2st (Figure 1A and Figure S1A). These results indicated that MeCP2 had the strongest binding affinity to itself and MBD2 and vice versa MBD2 exhibited the most prominent associations to itself and MeCP2.

We excluded MBD3 from our interaction studies and did not check for MeCP2 and MBD2 binding to MBD3, as MBD3 has been reported to be unable to bind to methylated DNA [4]–[6] and unable to exhibit strong accumulation at pericentric heterochromatin. We therefore ruled out that MBD3 could contribute to the aggregation of pericentric heterochromatin.

With the salt conditions (500 mM NaCl containing lysis buffer), used to extract the proteins from the cells for the *in vitro* pull down assays, one could not exclude that the observed interactions might be bridged to some extent by DNA. For that reason, we repeated the observed MeCP2 and MBD2 homo- and hetero-interactions this time using 1 M NaCl containing lysis buffer for the extraction of the proteins plus addition of ethidium bromide (EtBr) to disrupt potential protein-DNA interactions [35]. Incubation of recombinant RFP-tagged MeCP2 (MeCP2R) or RFP alone immobilized to sepharose-beads with GFP-fused full-length MeCP2 (MeCP2G) again showed a clear binding of MeCP2G to RFP-labelled MeCP2 but not to the RFP control (Figure 1B and Figure S1B). The specific MeCP2 homo-interaction could also be detected upon addition of ethidium bromide (EtBr), underlining that the observed direct binding was independent from DNA bridging (Figure 1B). Furthermore, we could detect specific binding of MBD2G to immobilized RFP-fused MBD2 (MBD2R) (Figure 1C and Figure S1B) as well as of MeCP2G to immobilized MBD2R (Figure 1D and Figure S1B) without and upon addition of EtBr.

Although hydrodynamic studies claimed that recombinant MeCP2 has the properties of a monomer [26], [27], the outcome of our *in vitro* pull down analyses showed that MeCP2 does directly interact with itself in the absence of DNA. These findings are in line with recent reports describing the formation of monomeric and dimeric foci of MeCP2 when bound to DNA [25]. We also show clear direct hetero-binding of MeCP2 with MBD2 as well as homo-interactions of MBD2 to itself, giving support to the hypothesis that MeCP2 and MBD2 homo- as well as hetero-interactions could indeed constitute one additional factor contributing to the clustering of pericentric heterochromatin *in vivo*.

### MeCP2 and MBD2 form Homo- and Hetero-interactions *in vivo*

**Figure 2:**
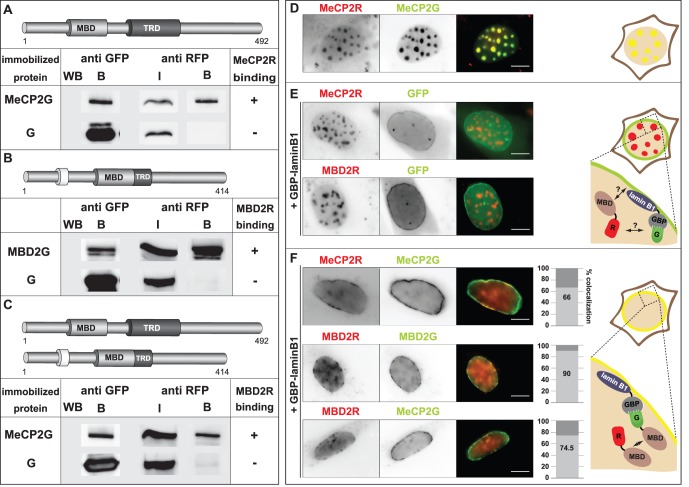
Homo and hetero-interactions of MeCP2 and MBD2 *in vivo*. (**A, B, C**) GFP alone and GFP- and RFP-tagged MeCP2 and MBD2 were co-expressed in HEK293-EBNA cells as indicated. After extraction, co-immunoprecipitation assays were performed at 137 mM (**A**) or 274 mM (**B** and **C**) NaCl using GBP-bound beads. The bound fraction (B) of the immobilized proteins used for the interaction assay was visualized by western blot using anti GFP antibody. The input (I) and bound fraction (B) of the interacting RFP-labelled proteins were visualized using anti RFP antibody. The input (I) represents 3.75% (**A**) and 7.5% (**B** and **C**) of the total reaction volume. (**A**) Homo-interactions of MeCP2, (**B**) binding of MBD2 to itself and (**C**) hetero-interactions of MeCP2 and MBD2. (**D, E, F**) C2C12 mouse cells were transfected with plasmids coding for (**D**) RFP and GFP-fused MeCP2, (**E**) RFP-fused MeCP2 or MBD2, GFP control and a protein fusion of the GFP binding protein (GBP) and lamin B1 (GBP-laminB1), (**F**) two fluorescently labeled methyl-cytosine binding domain (MBD) proteins as indicated and GBP-laminB1. Shown are representative images of mouse cells expressing the proteins as indicated. Scale bar: 5 µm. The graphs represent % of cells with co-localization of the fluorescent signals. The experiment was repeated twice, analyzing 100 cells (n = 100) each time. Right side: Schematic illustrations of the interaction assay. **(D**, right side**)** Localization of RFP- and GFP-fused MeCP2 proteins at pericentric heterochromatin in mouse cells. **(E**, right side**)** Mouse cell expressing RFP-labelled MBD protein, GFP control and GBP-laminB1. Due to GBP-laminB1, GFP is recruited at the lamina. The RFP-MBD protein is localized to heterochromatin. **(F**, right side**)** Mouse cells expressing GFP and RFP-tagged MBD proteins and GBP-laminB1. In case of an interaction between both fluorescently labeled MBDs, the RFP and GFP signals co-localize. G and R stand for GFP and RFP respectively.

To test, whether the observed interactions could be detected in mammalian cells, we co-transfected HEK 293 cells with plasmids coding for RFP- and GFP-fused MeCP2 as well as MeCP2R and GFP alone. The cells were lysed and the cell extract was subjected to immunoprecipitation using anti GFP antibody. After separation of the probes through SDS-PAGE, western blot analysis using anti RFP antibody showed that MeCP2R bound to MeCP2G but not to the GFP control (Figure 2A and Figure S2). The same procedure was repeated by co-transfection of HEK-293 cells with RFP- and GFP-fused MBD2 as well as MBD2R and MeCP2G and clearly indicated that MBD2 homo- and MBD2-MeCP2 hetero-interactions were observed in mammalian cells (Figure 2B and C and Figure S2).

In a next step, we aimed to visualize the observed *in vivo* interactions in single cells *in vivo*. For that reason we made use of a modified form of the fluorescent two-hybrid assay [36]. Instead of tethering the fluorescent MeCP2 or MBD2 protein to a chromosomal lac operator array, we artificially tethered GFP-labelled MeCP2 or MBD2 to the nuclear lamina. This was achieved by co-transfection of mouse C2C12 cells with plasmids coding for the GFP tagged MBD protein or GFP control alone and a fusion of the GFP binding protein (GBP) and lamin B1 [28]. Through this cellular nanotrap at the nuclear lamina, GFP or GFP fused proteins get recognized and bound by the GBP tethered to the lamina and get therefore additionally recruited to the lamina [28]. In the case of a triple transfection of the cells with plasmids coding for the fusion of GBP and lamin B1 (GBP-laminB1), a GFP-labelled bait and a RFP-fused prey protein, the bait is targeted to the lamina through its binding to GBP-laminB1. An interaction between the GFP-tagged bait and the RFP-fused prey and therefore the additional recruitment of the prey to the lamina can get visualized through co-localization of the GFP and RFP fluorescent signals (Figure 2E and F).

To exclude any binding of the two fluorescent tags with each other as well as recruitment of RFP-fused MeCP2 and/or MBD2 to the lamina, we triple transfected mouse cells with plasmids encoding GBP-laminB1, GFP control and RFP fused MeCP2 or MBD2 (Figure 2E). Whereas GFP alone was almost equally distributed along the lamina through its binding to GBP-laminB1, no co-localization of the RFP and GFP fluorescent signals was detectable, ruling out any binding of MeCP2R or MBD2R to the lamina or of RFP to the lamina, GFP alone or GBP-laminB1 (Figure 2E). The RFP- labeled MBD proteins further showed their expected localization to pericentric heterochromatin, comparable to mouse cells expressing MBD proteins in the absence of GBP-laminB1 (Figure 2D).

In a next step we wanted to examine, whether we could observe *in vivo* homo-interactions of MeCP2 in single cells using this method. Triple transfection of mouse cells with GBP-laminB1, MeCP2G and MeCP2R resulted in clear co-localization of both fluorescent proteins at several stretches along the lamina, visualizing binding between lamina tethered MeCP2G and MeCP2R (Figure 2F upper row). As the majority of ectopic fluorescently labeled MeCP2 was localized at the lamina, the common heterochromatic foci of fluorescent MeCP2 were nearly undetectable (Figure 2D and F). Besides, triple transfection of GBP-laminB1, MBD2G or MeCP2G and MBD2R further showed cells with clear co-localization of the RFP and GFP signal, indicating *in vivo* binding of MBD2 to itself (Figure 2F middle row) and of MBD2 to MeCP2 (Figure 2F lower row).

Using two independent *in vivo* assays, we clearly illustrate that the observed direct associations between MeCP2 and MeCP2, MeCP2 and MBD2 as well as MBD2 and MBD2 do also take place *in vivo* and could be visualized in single cells.

### Mapping Domains Responsible for MeCP2 and MBD2 Homo- and Hetero-interactions

**Figure 3:**
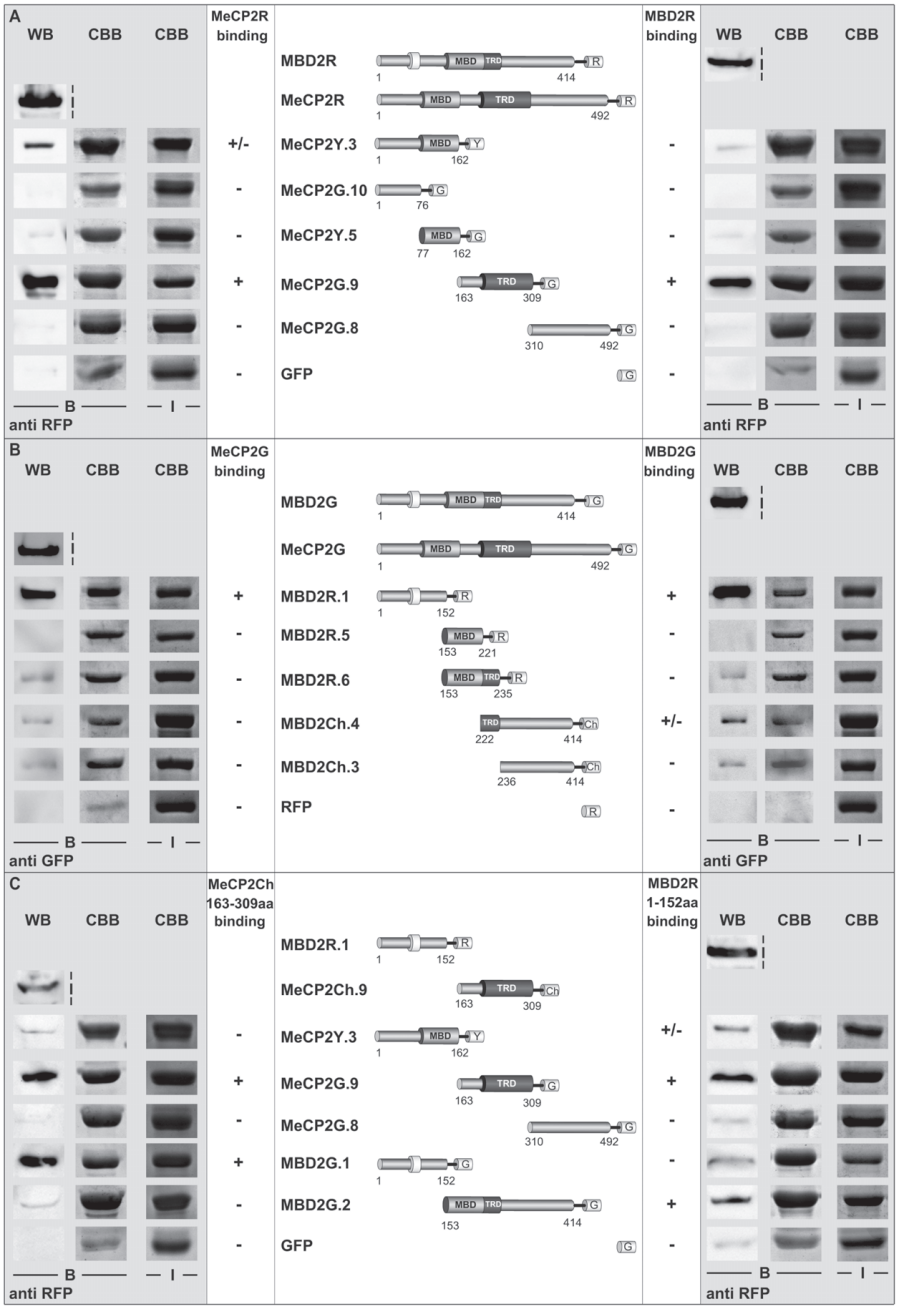
Mapping of domains responsible for MeCP2 and MBD2 homo- and hetero-interactions. (**A**) In vitro pull-down experiments with immobilized YFP- or GFP-fused MeCP2 constructs as illustrated and full-length (fl) RFP-labelled MeCP2 (MeCP2R) and MBD2 (MBD2R). The interactions were performed in PBS buffer supplemented with 125 mM NaCl and 0.05% NP-40. Interacting RFP-tagged proteins (B) were assessed by western blot with anti-RFP and Coomassie Brilliant Blue (CBB) staining of the gel after protein transfer was performed to visualize the immobilized YFP- or GFP-fused constructs (B). For input control (I), ¼ of the protein amounts used for the interaction assay was taken and stained by CBB or western blot using anti RFP. (**B**) Pull-down experiments using Cherry- (Ch) or RFP-fused MBD2 constructs as indicated, immobilized to RFP-binding protein (RBP) bound sepharose beads, and GFP-labelled fl MBD2 (MBD2G) and MeCP2 (MeCP2G). The assays were performed in PBS supplemented with 125 mM NaCl and 0.05% NP-40. The interacting proteins (B) were analyzed by western blot with anti GFP and CBB staining of the gel after protein transfer for the immobilized Cherry- or RFP-fused MBD2 constructs (B). As for (**A**), ¼ of the protein amounts used for the interaction assay were loaded as input control (I) and visualized either by western blotting with anti GFP or CBB respectively. (**C**) In vitro binding assays using YFP- or GFP-labelled MeCP2 or MBD2 constructs as indicated, immobilized to GFP-binding protein (GBP) bound beads, and RFP-fused MBD2 NH2-terminal domain (NTD) and Cherry-fused MeCP2 ID-TRD. The interaction was performed in PBS supplemented with 125 mM NaCl and 0.05% NP-40. Interacting Cherry- or RFP-tagged proteins (B) were assessed by western blot with anti RFP and Coomassie Brilliant Blue (CBB) staining of the gel after protein transfer was performed to visualize the immobilized YFP- or GFP-fused constructs (B). ¼ of the protein amounts used for the interaction assay were loaded as input control (I) and visualized either by western blotting with anti RFP or CBB. G, R and Ch stand for GFP, RFP and Cherry respectively.

After establishing that full-length MeCP2 and full-length MBD2 associate *in vitro* and *in vivo*, we asked, which domains could be responsible for the observed interactions. For that reason, we performed *in vitro* pull-down experiments with recombinant proteins extracted from Sf9 insect cells using 1 M NaCl containing lysis buffer to disrupt DNA binding of the proteins. After incubation of full-length MeCP2R or MBD2R proteins with equal amounts of GFP or YFP-labelled MeCP2 deletions immobilized to GBP bound sepharose beads, the protein complexes were separated by SDS-PAGE and analysed by western blot using anti RFP. This mapping of MeCP2 domains responsible for MeCP2 association to itself and to MBD2 revealed the region spanning MeCP2 interdomain (ID) and TRD (ID-TRD, amino acids 163–309) to directly interact with MeCP2R and MBD2R to the strongest extent, whereas other MeCP2 domains showed very weak to no binding (Figure 3A and Figure S3A). Subsequent mapping of RFP/Cherry-labeled MBD2 domains to full-length MeCP2G and MBD2G showed, that the NH_2_-terminal domain of MBD2 (NTD, amino acids 1–152) exhibited strong binding to both MeCP2 and MBD2. Binding to full-length MBD2 of those domains of MBD2 located COOH-terminal to the NTD, was very weak compared to the one of the NTD (Figure 3B and Figure S3B).

**Figure 4:**
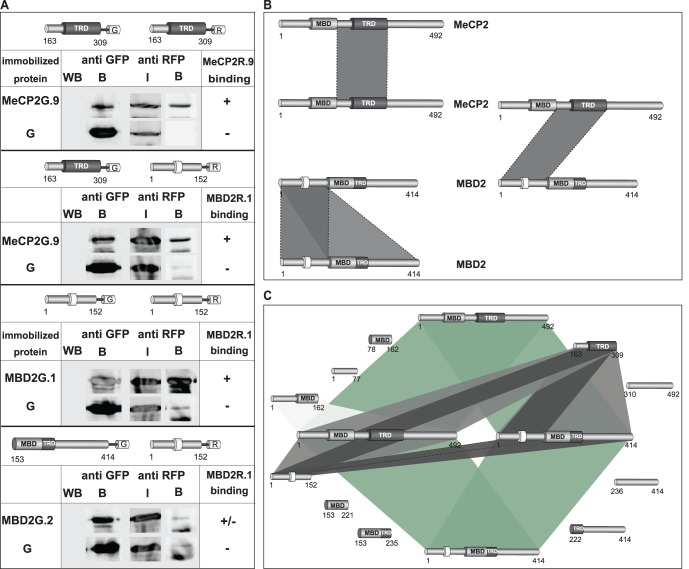
*In vivo* homo- and hetero-interactions between domains of MeCP2 and MBD2. (**A**) GFP- and RFP-tagged domains of MeCP2 and MBD2 as well as GFP control were co-expressed in HEK293-EBNA cells as indicated. After cell lysis using 200 mM NaCl buffer conditions, the extract was incubated with GBP-bound beads for co-immunoprecipitation analysis under the same buffer conditions. The immobilized protein complexes were washed afterwards with the same buffer as used for lysis and co-immunoprecipitation. The immobilized GFP-labeled proteins (B) used for the interaction assay were visualized by western blot using anti GFP antibody. The input (I) and the co-immunoprecipitated fraction (B) of the RFP-labeled proteins were visualized through western blot using anti RFP antibody. The input (I) represents 7% of the total reaction volume. (**B** and **C**) Schematic representation of the domains responsible for the homo-and hetero-interactions of MeCP2 and MBD2 (dark grey) illustrating the outcome of the *in vivo* and *in vitro* interaction analyses. Numbers stand for amino acid (aa) coordinates. (**C**) Full-length (fl) MeCP2 and MBD2 directly bind to themselves and each other (green). In case of the MeCP2 homo-interaction, the ID-TRD (aa 163–309) is the domain of MeCP2 that mediates strong direct binding to fl MeCP2 (light grey) and further recognizes the ID-TRD domain independently (dark grey). Regarding MeCP2 and MBD2 hetero-interaction, MeCP2 ID-TRD domain exhibits strong association to fl MBD2 in comparison to other MeCP2 domains (light grey) and further directly and independently interacts to the NH_2_-terminal domain (NTD, aa 1–152) of MBD2 (dark grey). The NTD is also the only domain of MBD2 that shows strong binding to fl MeCP2 (light grey) and strongly binds to MeCP2 ID-TRD independently (dark grey). In the case of the MBD2 homo-interaction, the NTD is again the region of MBD2 exerting the strongest binding to fl MBD2 (light grey) and further recognizes MBD2 NTD and COOH-terminal domain (dark grey).

In summary, our analyses showed MeCP2 ID-TRD and MBD2 NTD as the important domains for MeCP2 and MBD2 homo- and hetero-interactions (Figure 3A and B). Consequently, we addressed, whether the ID-TRD of MeCP2 alone binds to the ID-TRD of MeCP2 and NTD of MBD2 and whether MBD2 NTD alone preferentially associates with ID-TRD of MeCP2 and NTD of MBD2. *In vitro* pull-down experiments using Cherry/RFP-labeled ID-TRD or NTD and immobilized GFP/YFP-fused domains of MeCP2 and MBD2 showed, that ID-TRD exhibited the strongest binding to itself and MBD2 NTD compared to MeCP2 COOH-terminus and the region comprising NH_2_-terminus plus MBD and MBD2 COOH-terminus (amino acids 153–141) (Figure 3C and Figure S4A). MBD2 NTD alone further showed the strongest affinity to the ID-TRD of MeCP2 and the COOH-terminus of MBD2 (Figure 3C and Figure S4A). That MBD2 NTD interacted with MBD2 COOH-terminus stronger than to the MBD2 NTD domain itself suggested an additional head-to-tail aggregation. In a subsequent step, we further tested whether the homo- and hetero-associations of MeCP2 and MBD2 specific domains could also be observed *in vivo* performing co-immunoprecipitation analysis. For that, HEK 293 cells were co-transfected with plasmids coding for RFP- and GFP-fused ID-TRD, MBD2 NTD and COOH-terminus. After lysis, the cell extract was subjected to immunoprecipitation using GBP protein coupled to beads. The protein complexes were then separated by SDS-PAGE and analyzed by western blot using anti RFP antibody. Whereas only low amount of the RFP-fused proteins could be detected bound to the GFP alone control, we could observe specific homo-interactions between RFP- and GFP-labeled ID-TRD of MeCP2 as well as of MBD2 NTD (Figure 4A and Figure S4B). Furthermore, *in vivo* associations between MeCP2 ID-TRD and the NTD of MBD2 were evident. Whereas the *in vitro* experiments (Figure 3C and Figure S4A) indicated an interaction between the NTD and the COOH-terminus of MBD2 that was more prominent than the homo-association of MBD2 NTD, our co-immunoprecipitation analyses clearly showed stronger binding of MBD2 NTD to itself than to the COOH-terminus (Figure 4A and Figure S4B). To estimate the strength of these interactions, we further repeated the co-immunoprecipitation analyses using 200 mM NaCl containing buffer and this time washed the protein complexes with buffer supplemented with 300 mM NaCl (Figure S4C). Whereas especially the homo-interaction between MBD2 NTD was still detectable, no clear association was observed between ID-TRD with itself as well as with the MBD2 NTD and the NTD and COOH-terminus of MBD2.

Based on these interaction studies, we show that MeCP2 directly mediates interactions to itself and MBD2 via its ID-TRD. We identified the NTD as the domain of MBD2 responsible for its direct binding to MeCP2. The NTD of MBD2 is further capable to form associations with full-length MBD2 (Figure 4B and C). We can exclude that the observed interactions were bridged by DNA, as extraction of all proteins was performed at 1 M NaCl lysis conditions to disrupt potential protein-DNA association.

Our data favor a mechanism for MeCP2-induced interconnection of nucleosomes involving MeCP2 homo-associations, mostly through the ID-TRD. Furthermore, hetero-association of MeCP2 with other chromatin-bound MBD proteins could cause and stabilize MeCP2-mediated heterochromatin aggregation as can be seen from the association between MeCP2 and MBD2 *in vitro* and *in vivo*. We show both, MeCP2 direct binding to MBD2, as well as the independent interaction of the ID-TRD of MeCP2 with full-length MBD2 and MBD2 NTD. We also observed strong direct binding of MBD2 with itself. The observed hetero-interactions of MeCP2 and MBD2 further give rise to the assumption, that a multitude of homo- and hetero-associations between the MBD proteins could coordinate heterochromatin reorganization *in vivo*. This is supported by the fact that, except for MBD3, all MBD proteins are localized at pericentric heterochromatin and mostly MBD2 and MeCP2 are capable of inducing dose-dependent chromatin aggregation [7]. Functional redundancy between the MBD proteins has been suggested based on the finding that clustering of pericentric heterochromatin is maintained in MeCP2-deficient mouse tissues [7]. Moreover, our findings could suggest overlapping functions as a result of cross-interactions, which in all probability mediate and stabilize chromatin aggregation.

It has recently been proposed that MeCP2 is organized into a NH_2_-terminal part consisting of the MBD and its flanking regions (amino acids 1–75; ID: amino acids 164–210) that exert modulating and stabilizing effects on MBD DNA binding *in vitro* [24]. The second unit is shown to be formed by TRD and the CTD that can independently induce chromatin compaction and intra-associations of nucleosomal arrays [24]. As a higher ratio of TRD-CTD is required to induce chromatin clustering comparable with full-length MeCP2 *in vitro*, synergy between both units has been suggested to underlay full MeCP2 function regarding DNA binding and chromatin clustering [24]. These findings underscore the possibility that MeCP2 requires its MBD domain to be able to accumulate at chromatin and to induce a certain level of chromatin aggregation. The ID-TRD domain could in addition - based on our experiments - exert cross-linking potential, which might increase the overall heterochromatin clustering ability of MeCP2. In this regard, we have recently ectopically targeted to heterochromatin a MeCP2 Rett mutant with a missense mutation within the MBD and thus unable to bind methylated cytosines and could observe a rescue of its heterochromatin clustering ability [21]. We propose that the function of the MBD proteins in shaping chromatin higher order structure relies on multiple DNA, chromatin, chromatin-protein interactions and homo- and hetero-associations between MBDs further enhance this chromatin web.

## Supporting Information

Figure S1 **Overview of uncut gels and membranes for** **Figure 1A, 1B, 1C and 1D** **.** Input cell extract (I) and bound fraction (B). Numbers on the marker bands indicate the protein size in kilodalton (kD). Whole membranes and stained gels (CBB) are shown in **(A)** for Figure 1A and in **(B)** for **Figure 1B, 1C** and 1D.
(EPS)

Figure S2 **Overview of uncut membranes for** **Figure 2A, 2B and 2C** **.** Input cell extract (I) and bound fraction (B). Lanes, that were on the original blot not next to each other, were moved together for facilitated understanding which is indicated by dashed lines. Numbers on the marker bands indicate the protein size in kilodalton (kD).
(EPS)

Figure S3 **Overview of uncut gels and membranes for** **Figure 3A and B** **.** Input cell extract (I) and bound fraction (B). Numbers on the marker bands indicate the protein size in kilodalton (kD). Dashed lines indicate that membrane/gels were assembled together, as they were originally not next to each other or come from different membranes and/or gels. Uncut stained gels (CBB) and membranes are shown in **(A)** for Figure 3A and in **(B)** for **Figure 3B**.
(EPS)

Figure S4 **Overview of uncut gels and membranes for** **Figure 3C** **and** **Figure 4A:** **Protein domains responsible for MeCP2 and MBD2 homo- and hetero-interaction.** Input cell extract (I) and bound fraction (B). Numbers on the marker bands indicate the protein size in kilodalton (kD). Whole stained gel (CBB) and membrane pictures are shown in **(A)** for Figure 3C and in **(B)** for Figure 4A. Dashed lines indicate that membrane/gels pieces were assembled together, as they were originally not next to each other or come from different membranes and/or gels. **(C)** Co-Immunoprecipitation analysis was done as for Figure 4A (Figure S4B) with the exception, that after co-immunoprecipitation, the protein complexes were washed with buffer containing 300 mM NaCl. HEK293-EBNA cells were co-transfected with plasmids coding for GFP- and RFP-tagged domains of MeCP2 and MBD2 as well as GFP control. After cell lysis using 200 mM NaCl containing buffer, co-immunoprecipitation was performed by incubation of the cell extract with GBP-bound beads. The immobilized protein complexes were washed afterwards with buffer containing 300 mM NaCl. The immobilized GFP-labeled proteins (B) used for the interaction assay were visualized by western blot using anti GFP antibody. The input (I) and the co-immunoprecipitated fraction (B) of the RFP-labeled proteins were visualized through western blot using anti RFP antibody. The input (I) represents 7% of the total reaction volume.
(EPS)
